# Cognitive impairment and the associated factors among women with a history of pregnancy complications in rural southwestern Uganda

**DOI:** 10.1371/journal.pone.0293258

**Published:** 2023-10-31

**Authors:** Raymond Bernard Kihumuro, Peace Kellen, Sarah Chun, Edith K. Wakida, Celestino Obua, Herbert E. Ainamani

**Affiliations:** 1 Department of Psychiatry, Mbarara University of Science and Technology, Mbarara, Uganda; 2 Office of Research Administration, Mbarara University of Science and Technology, Mbarara, Uganda; 3 Department of Medicine, California University of Science and Medicine, Northridge, California, United States of America; 4 California University of Science and Medicine, Northridge, California, United States of America; 5 Department of Pharmacology and Therapeutic, Mbarara University of Science and Technology, Mbarara, Uganda; 6 Department of Mental Health, Kabale University School of Medicine, Kabale, Uganda; University of the Witwatersrand Johannesburg, SOUTH AFRICA

## Abstract

**Background:**

Worldwide, there is a growing concern about the rising number of people with declining cognitive functioning. However, findings on this phenomenon are inconclusive. Our study aimed to assess the prevalence of cognitive impairment and the associated factors in women with a history of pregnancy complications in rural southwestern Uganda.

**Methods:**

This was a cross-sectional study carried out among women above 40 years of age in the greater Kabale district of southwestern Uganda between March and April 2022. Study participants were identified using a consecutive sampling method. Predictor variables included pregnancy complications and other social demographic factors that were assessed by semi-structured interviews while cognitive functioning as an outcome variable was assessed by Montreal Cognitive Assessment (MoCA-B) tool. Data were analyzed using STATA at a 95% Confidence level. Logistic regression analyses were selected for statistical modelling while odds ratios were calculated to assess the strength of associations between the predictor and outcome variables.

**Results:**

In total, 75% (212/280) of participants had some form of cognitive impairment, with 45% (123/280) falling into mild CI, 31% (86/280) moderate CI and 4% (10/280) severe CI. Twenty-three percent (68/280) of participants fell into category of normal cognitive functioning. Participants with >65 years of age had higher odds of developing cognitive impairment (OR = 2.94; 95%CI: 0.96–9.04, p = 0.06) than those with < 65 years of age. Protective factors to cognitive impairment include delivering from a health facility (OR = 0.31,95% CI:0.16–0.60, p = < .001), primary and post primary levels of education (OR = 0.05; 95% CI: 0.02–0.13, p<0.001, OR = 0.04; 95%CI: 0.02–0.23, p<0.001) respectively.

**Conclusion:**

Results from this study show a high prevalence of cognitive impairment among women with a history of pregnancy complications in rural southwestern Uganda. Interventions geared toward preventing cognitive impairment among females with a history of pregnancy complications should be emphasized.

## Background

In recent years, there has been a worldwide growing concern about the rising number of people with declining cognitive functioning and dementia [1]. In 2019, the global estimates for people with cognitive impairment were at 57.4 million people [2]. Out of this number, 60% were found to be residing in low- and middle-income countries including Africa [3, 4]. Surprisingly, Sub-Saharan Africa accounts for the largest burden of cognitive impairment [5]. A recent study in southwestern Uganda found that 56% of the participants with a history of traumatic brain injury screened positive for cognitive impairment [6]. While another study in southwestern Uganda found the prevalence of cognitive decline at 20% among adults above 60 years of age in the general population [7]. Various factors such as low educational attainment, midlife hypertension, midlife obesity, hearing loss, late-life depression, diabetes, physical inactivity, smoking, social isolation, and genetic influences seem to pose a risk for this high prevalence of cognitive deterioration [1, 7–10]. Numerous studies have indicated that the prevalence of cognitive impairment in women is disproportionately higher compared to men [11–13]. The high prevalence of cognitive impairment and dementia in women could be linked to the biological changes and events around pregnancy which occur in women only. Hence, suggesting the possible role of reproductive history on the risk of later dementia [14–20]. Previous studies have shown a higher risk of developing cognitive impairment and dementia in postmenopausal women [21–23]. While others have reported varying results on the association between different pregnancy complications such as pre-eclampsia and cognitive impairment later in life [14, 24–26].

A retrospective cohort study in the United States that assessed women on cognitive domains of psychomotor speed, executive functions, and memory, suggested that hypertension in pregnancy did not independently influence neurocognitive impairment later in life [27]. Furthermore, a study in China reported increased risk of cognitive impairment among women in the postpartum period [28]. Similarly, a systematic review that summarized studies investigating an association between a history of preeclampsia and cognitive function later in life, highlighted that the association between hypertensive disorders in pregnancy and cognitive impairment using standard neuro-cognitive tests was unclear [29]. This study pointed out the need for further studies to fill the gap in the knowledge on the association between pre-eclampsia and cognitive outcomes.

Literature, mainly from high-income countries, shows that cognitive impairment is more prevalent in women than men but is inconclusive on the association between various pregnancy complications and later cognitive decline. However, even though there is conflicting literature on this phenomenon mainly from high income countries, there is a paucity of research in Africa and more especially in Uganda. Most previous studies have examined cognitive impairment among children [30], refugees [31] and people living with HIV, but not women with a history of pregnancy complications [32, 33]. This lack of sufficient data on cognitive functioning in low resource countries among women limits the formulation of policies and interventions to modify risk factors such as pregnancy complications.

In this study, we aimed to assess the occurrence of cognitive impairment and the associated factors among the women with a history of pregnancy complications in rural southwestern Uganda. Moreover, previous studies in southwestern Uganda have shown a relatively high prevalence of dementia and cognitive impairment [7, 12]. In this study and the rest of manuscript, pregnancy complications will refer to hypertensive disorders during pregnancy such as pre-eclampsia, stillbirth and miscarriage.

## Methods

### Study design and setting

This was a quantitative cross-sectional study carried out in March and April 2022 among women aged 40 years and above with a history of pregnancy complications in districts of Kabale, Rubanda and Rukiga where the prevalence of dementia is presumed to be high [7, 34]. This area has a high prevalence of people with Alzheimer’s disease, other related dementias [7, 12, 35] and high fertility rate [36]. We excluded women who were less than two years postpartum, the very ill, and those who were known to have been diagnosed with severe mental illness. The reason for excluding women who were less than two years postpartum was to help us capture long-term cognitive effects of pregnancy complications and mitigate the effect of postpartum depression that could exist among the mothers [37, 38]. In this study,"the very ill" referred to women who were experiencing severe acute illnesses or were admitted to the hospital for a medical condition unrelated to pregnancy complications. This exclusion criterion was implemented to minimize the impact of acute illness on cognitive function and ensure that the observed cognitive impairment was primarily associated with pregnancy complications rather than temporary illness-related.

### Sample size determination

A sample size of 278 participants was determined following the sample selection methods used by Hulley, Cummings, Browner, Grady, Newman [39]. n = N*X / (X + N– 1), where, X = Z_α/2_^2^ *p*(1-p) / MOE^2^, and Z_α/2_ is the critical value of the normal distribution at α/2 (e.g. for a confidence level of 95%, α = 0.05, and the critical value is 1.96). MOE is the margin of error, p is the sample proportion, and N is the population size.

### Ethical consideration

Ethical approval was obtained from the Mbarara University of Science and Technology Research Ethics Committee (MUST-REC) with ethical approval number MUST-2021-325. Additionally, we acquired administrative clearance from the district administration and the local leaders from the two Districts of Kabale and Rubanda. Before the interview, content, procedure, risks, the right to withdraw, and confidentiality were explained to each participant and a written informed consent (signature or fingerprints) of the participants were obtained.

### Data collection and sampling procedure

We first contacted the District Health Officers (DHOs), who in turn connected the research team to the Village Health Teams (VHTs) who helped us access potential participants. In Uganda, the VHTs are the first line health workers that monitor and take care of the patient’s records at a village level [40].

Using consecutive sampling method, we collected data from 280 women with a history of pregnancy complications between March and April of 2022. We excluded women who were unable to provide information due to physical or cognitive challenges such as deafness/mutism or acute intoxication. Two medical doctors and one Psychologist collected the data. The three research assistants went through a three-day training in neuro-cognitive assessment and practiced the assessment before they administered the interviews. During the training, we conducted role plays to accomplish high inter-rater reliability [41]. In addition, following the requirement of use by the validators of MoCA-tool [42], the principle investigator (RBK) received a free online training on the use of MoCA-B in dementia screening and assessment.

Face to face interviews with the participants lasted between 45–60 minutes in a private setting within the health center premises. Women with severe symptoms of cognitive impairment were referred to the nearest health facilities for further management. All interviews were conducted in Runyankore Rukiga, a traditional local language spoken by people in southwestern Uganda.

## Data collection tools

The first part of our instruments included social demographic characteristics (age, level of education, marital status), history of alcohol use, family history of dementia, HIV status, history of pregnancy complications i.e., hypertensive disorders in pregnancy, still birth, and miscarriage, age of at first pregnancy as the predictor variables. All instruments were translated into Runyankore Rukiga and back-translated to English in a blind written form as recommended by previous studies [43].

### Primary outcome variable

Information regarding cognitive impairment as the primary outcome variable was gathered using the Montreal Cognitive assessment tool which assessed the presence of cognitive impairment and dementia (MoCA) [8]. There are two versions of the MoCA tool; MoCA and MoCA-B. In our study, we utilized an adapted version of the Montreal Cognitive Assessment-Basic (MoCA-B) tool. In consultation with a Clinical Psychologists on our team, the adaptation of the MoCA tool was based on a previous study conducted in the same setting by Kintu and colleagues [6]. The adaptation involved making culturally relevant changes to the original MoCA-B tool, such as replacing currency references from dollars to shillings, and animal references from tiger to lion, peacock to hen, and zebra to cow. These modifications were made to ensure that the tool is culturally sensitive and more applicable to our specific sample.

By adopting the adapted MoCA-B, we aimed to enhance the relevance and cultural appropriateness of the cognitive assessment for the population under study. This approach acknowledges the importance of considering cultural factors when assessing cognitive functions in different settings.

The MoCA tool is a rapid screening instrument which assesses cognitive domains of orientation, short-term memory, visuo-spatial abilities, attention/concentration, language, and aspects of executive functioning.

This tool possesses strong psychometric properties with good test-retest reliability and internal consistency (0.83) [44, 45], and has been validated in sub-Saharan Africa i.e., in South Africa [45] and in East Africa [44]. This version was specifically created for screening patients with low education and low literacy levels [45]. The total score for MoCA-B tool ranges from 0–30 suggesting with a cutoff point >25 suggesting normal cognitive functions, moderate CI (18–25), mild (11–18) and <10 as severe cognitive impairment.

## Data analysis and management

Data were secured in a cabinet under lock and the key was only accessible by the lead investigator. Data were reviewed for completeness, missing fields identified and filled at the end of each data collection day. We generated data into excel spread sheet using Kobo Toolbox (Harvard Humanitarian Initiative, Cambridge, Massachusetts, United States of America). From excel, cleaned data was entered into STATA 17.0 (Stata Corp, College Station, Texas, USA). We computed standard statistics to summarize characteristics of the sample as well as the prevalence of dementia within our sample based on the previous studies [45]. For purposes of examining the association between various social demographic categories and cognitive functioning, we dichotomized our sample into two categories >25 suggesting normal cognitive functioning and >25 cognitive impairment. This estimate was analyzed using logistic regression.

## Results

### Participant’s social demographic characteristics

Overall, 280 women aged between 40 to 90 years with a mean age of (53.5[10.6]) participated in this study. Nearly all the participants in this study were married 83.6% (234/280). Almost a quarter of our participants 29% (80/280) had experienced more than one pregnancy complication See Table 1.

**Table 1:** Showing descriptive statistics of the participant’s characteristics (n = 280).

Variables	Frequency	Percentage (%)
**Bleeding**		
No	222	79
Yes	58	22
**Hypertensive disorders**		
No	265	95
Yes	15	5
**Miscarriage**		
No	73	26
Yes	207	74
**Still birth**		
No	238	85
Yes	42	15
**Pregnancy complications**		
< = 1	200	71
>1	80	29
**Education**		
Primary education	245	88
Post Primary education	35	12
**Marital status**		
Others	46	16
Married	234	84
**HIV status**		
Negative	234	84
Positive	46	16
**Place of delivery**		
Other	106	38
Hospital	174	62
**Age, Years**		
40–65	240	86
Above 65	40	14
**Age at first pregnancy, Years**		
< = 16	37	14
17–21	132	50
22–26	69	26
> = 27	27	10
**Cognitive function Categories**		
Normal	68	23
Mild impaired	123	45
Moderate Impaired	86	31
Severe Impaired	10	4

### Occurrence of cognitive impairment among women with a history of pregnancy complications

In total, 75% (212/280) of our participants had some form of cognitive impairment with 45% (123/280) falling into mild CI, 31% (86/280) moderate, 4% (10/280) severe CI, and 23% (68/280) of participants fell into the category of normal cognitive functioning (see Table 1).

## Factors associated with cognitive functioning among women with a history of pregnancy complications

To estimate for the strength of different factors that were associated with cognitive functioning, multiple logistic regression between were used. At a bivariate level (Unadjusted analysis) results revealed that place of delivery, age of women and education level had a statistically significant association with cognitive functioning (p<0.05).

At a multivariate level (Adjusted analysis), results revealed that participants who delivered from health facilities had less chances of developing cognitive impairment compared to those who delivered from other places other than a health facility (OR = 0.31,95%CI:0.16–0.60, p = < .001). Participants who were more than 65 years of age, had substantially high odds of developing cognitive impairment compared those who are 65 and below (OR = 2.94; 95%CI: 0.96–9.04, p = 0.06).

Participants with primary and post primary as their highest level of education had lower chances of developing cognitive impairment compared to those with no formal education (OR = 0.05; 95% CI:0.02–0.13, p<0.01 and OR = 0.04; 95% CI:0.02–0.23, p < .001) (Table 2).

**Table 2:** Bivariate and multivariate logistic regression of pregnancy complications associated with cognitive functioning.

	OR	S.E	P_value	95% CI		AOR	S.E	P_value	95% CI	
**Place of delivery**										
Other Places	Ref									
Health Facility	0.23	0.08	<0.001	0.11	0.47	0.31	0.10	<0.001	0.16	0.60
**Current Age, years**										
40–65	Ref									
above 65	3.61	2.21	0.04	1.08	12.0	2.94	1.68	0.06	0.96	9.04
**Bleeding in pregnancy**										
No										
Yes	2.03	1.12	0.19	0.69	5.96					
**Hypertension**										
No	Ref									
Yes	1.72	1.46	0.52	0.33	9.09					
**Miscarriage**										
No	Ref									
Yes	0.71	0.39	0.53	0.25	2.06					
**Stillbirth**										
No	Ref									
Yes	0.82	0.44	0.71	0.29	2.34					
**Pregnancy Complications**										
< = 1	Ref									
>1	1.58	0.55	0.18	0.80	3.13					
**Age at First Marriage**										
< = 16	Ref									
17–21	0.39	0.24	0.12	0.12	1.28					
22–26	0.41	0.26	0.15	0.12	1.41					
> = 27	0.44	0.31	0.25	0.11	1.77					
**Marital Status**										
Others	Ref									
Married	1.09	0.50	0.85	0.44	2.66					
**HIV Status**										
Negative	Ref									
Positive	0.71	0.31	0.44	0.30	1.68					
**Education Level**										
No Formal Education	Ref									
Primary	0.06	0.03	<0.001	0.02	0.15	0.05	0.02	<0.001	0.02	0.13
Post primary	0.09	0.05	<0.001	0.03	0.28	0.07	0.04	<0.001	0.02	0.23

## Discussion

The primary aim of our study was to assess the prevalence of cognitive impairment and the associated factors among women with a history of pregnancy complications. Our results indicate a high prevalence of cognitive impairment among the women with history of pregnancy complications in rural southwestern Uganda. Specific forms of cognitive impairment observed in our sample included; mild (45%), moderate (31%) and severe (4%). Participants above the age of 65 had greater odds of developing cognitive impairment than those below the age of 65, while giving birth from a health facility and having higher education positively correlated with cognitive functioning.

The prevalence of cognitive impairment in our study, is slightly higher than the study of Kintu and colleagues in southwestern Uganda which focused on urban residents with high literacy levels following traumatic brain injury [6]. Our results are also in line with a study done among elder women in rural China [46].

One possible reason for this high prevalence of cognitive impairment is that previous studies with low prevalence used different tools other than the MoCA-B to asses cognitive decline [7, 47]. While other studies were not clear on the methods used in assessing cognitive functions [18]. Another possible explanation for the high prevalence in our sample is that the prevalence of cognitive decline and dementia in southwestern Uganda where our sample was derived is reported to have high baseline levels [6, 7, 12].

The variation between our findings and findings from other previous studies could be due to the fact that these studies recruited participants who were older than 65 years and hence did not cater for the possibility of early onset of cognitive impairment [48]. The question that remains unanswered therefore, is whether the high prevalence of cognitive impairment is related to the history of pregnancy complications or whether there are other moderating factors that need to be considered. We therefore suggest that the specific aspects of risk and resilience of early onset of dementia in the context of African rural setting be investigated more closely in future studies.

In line with previous studies, we found an association between increased age and cognitive impairment [46]. The finding that increasing age is associated with cognitive impairment is not surprising. For example, a recent study from western Uganda done among survivors of traumatic brain injury revealed a positive correlation between older age and cognitive impairment [6]. While results from another study carried out by Mubangizi and colleagues in the same region, observed that the odds of developing dementia increased with an increase in age with in their sample [7].

One important finding in our study was the significant positive association between higher educational levels and cognitive functioning. We found that participants within our sample with seven and above years of education performed better on cognitive sections of the MoCA-B than those with lower education. Our finding about the correlation between education and cognitive performance is further confirmed by other studies that have shown educational enrichment in early life to acts as a defense against the development of cognitive impairment in late life [49, 50]. Similarly, our results confirm the cognitive reserve hypothesis which seems to propose that education has a protective effect that buffers cognitive impairment in the general population [51, 52]. For example, one study found out that cognitive reserve delayed decline in cognitive functions among the old people before the onset of Alzheimer’s disease [51]. We propose that the area of cognitive reserves in the form of enhancing education levels be emphasized by clinicians and policy makers.

The finding that having delivered babies from a health facility positively correlated with good cognitive functioning could be supported by previous studies which propose that women who deliver from home get less medical and health intervention that exposes them to a number of health deficiencies [53]. Interestingly, research has also shown that delivering from home increases the risk of children’s cognitive impairment in later adulthood [54].

Furthermore, our study population was largely comprised of women with low literacy levels. Low literacy levels were the biggest modifiable risk factor for development of cognitive impairment according to a systematic review in sub-Saharan Africa that looked for the risk factors of cognitive decline in the region [55]. We suggest that the likelihood of developing dementia among women with a history of pregnancy complications in low- and middle- income countries be explored further to understand the possible confounders and other predicator variables.

### Limitations

Interpretation of our findings is subject to certain limitations. First, the sample was restricted to women with a history of pregnancy complications and may not be generalized to the general population samples. A second important limitation of our study is the cross-sectional design which may not allow for causal conclusions. Thirdly, we also note that although the MoCA-B tool used to assess cognitive impairment in our study has been validated in other African countries [56], it has not been specifically validated in Uganda. We propose that future studies using the MoCA-B tool could validate it for use in Uganda.

Lastly, certain biases, such as recall and social desirability biases, common to retrospective designs may have affected the study findings, however, it is inevitable that such designs may be used for this kind of study.

## Conclusion and recommendations

In this cross-sectional study of women with a history of pregnancy complications, in rural southwestern Uganda, we found high prevalence of cognitive impairment. These findings suggest the need for further research into this phenomenon to ascertain the true picture in the population. Future studies could assess the effect of other confounders such as age at first pregnancy or first live birth, termination of pregnancy history, menopausal status and age at menopause, and iatrogenic menopause. Additionally, the comparative study between women with a history of pregnancy complications and those without is recommended to bring this phenomenon of cognitive impairment into focus.

## Supporting information

S1 Checklist STROBE statement—Checklist of items that should be included in reports of *cross-sectional studies*. (DOC)

S2 Checklist STROBE statement—Checklist of items that should be included in reports of *cross-sectional studies*. (DOC)

S1 Data set (XLS)

## Abbreviations

ADRD: Alzheimer’s disease and other related dementias
LAMICs: Low- and middle-income countries
MoCA-B: Montreal Cognitive Assessment-Basic
VHTs: Village Health Teams
