# Supplementary material for: Cognitive impairment and the associated factors among women with a history of pregnancy complications in rural southwestern Uganda
Source: PLoS One. 2023 Oct 31;18(10):e0293258. doi: 10.1371/journal.pone.0293258 (PMC10617700; doi:10.1371/journal.pone.0293258)
Supplement: S1 Checklist — (DOC) [file pone.0293258.s001.doc]

STROBE Statement—Checklist of items that should be included in reports of ***cross-sectional studies***

|  | Item No | Recommendation | Page number |
| --- | --- | --- | --- |
| **Title and abstract** | 1 | (*a*) Indicate the study’s design with a commonly used term in the title or the abstract: **Our study used a cross-sectional study design-this is reflected in the abstract** | **2** |
|  |  | (*b*) Provide in the abstract an informative and balanced summary of what was done and what was found. **Response:** **We have provided this information in the abstract and is reflected on page 2 and 3** |  |
| Introduction | | |  |
| Background/rationale | 2 | Explain the scientific background and rationale for the investigation being reported: Response: **Globally, there is a growing concern with a growing number of people with cognitive decline. Global statics show that this number has increased in Africa. However, studies in this phenomenon are mostly in HICs and almost none in East Africa. To increase the knowledge of the practitioners and enhancing policy in this area of concerned, research is needed. More information on this section is on page 3, 4 and 5** | **3,4 &5** |
| Objectives | 3 | State specific objectives, including any prespecified hypotheses: Response  **We aimed to assess the prevalence of cognitive impairment and the associated factors among the women with a history of pregnancy complications in rural southwestern Uganda.**  **We hypothesis that; a) the prevalence of cognitive impairment among the women with a history of pregnancy complications is high. b) different factors would be associated with cognitive impairment among the women with a history of pregnancy complications.** | **5** |
| Methods | | |  |
| Study design | 4 | Present key elements of study design early in the paper  Response: **This was a cross sectional study design utilising quantitative methods of data collection. More details are on page 5** | **5** |
| Setting | 5 | Describe the setting, locations, and relevant dates, including periods of recruitment, exposure, follow-up, and data collection.  Response: **The study carried out in March and April 2022 in the three neighbouring districts of Kabale, Rukiga and Rubanda in rural Southwestern Uganda. The country’s population is young with a big percentage of the population below 18 years old; The study was conducted 104 among women aged 40 years and above with a history of pregnancy complications in districts of Kabale, Rubanda and Rukiga where the prevalence of dementia is high [7, 34]. These districts in which the study was carried out are approximately 408km drive from Kampala city. This area has a high prevalence of people with Alzheimer's disease and other related dementias as documented by previous studies and high fertility rate. We excluded women who were less than two years postpartum, the very ill, and those who were known to have been diagnosed with severe mental illness.** | **5** |
| Participants | 6 | 1. Give the eligibility criteria, and the sources and methods of selection of participants: Response: **We excluded women who were unable to provide information due to physical or cognitive challenges such as deafness/mutism or acute intoxication. Women with a history of pregnancy complications were excluded. The Village health teams directed us to the women that were known of having had pregnancy complications.** | **6** |
| Variables | 7 | Clearly define all outcomes, exposures, predictors, potential confounders, and effect modifiers. Give diagnostic criteria, if applicable. **Response: Primary outcome variable Information regarding cognitive impairment as the primary outcome variable was gathered using the Montreal Cognitive assessment tool which assessed the presence of cognitive impairment and dementia (MoCA) [8]. There are two versions of the MoCA tool; MoC-A and MoCA-B. In this study, we adopted MoCA-B due to its cultural sensitivity and more relevant to our sample. The MoCA-B is a rapid screening instrument which assesses cognitive domains of orientation, short-term memory, visuo-spatial abilities, attention/concentration, language, and aspects of executive functioning.** Predictor **variables included. included social-demographic factors such as current age, level of education, number of pregnancies, age at first pregnancy, other pregnancy complications of miscarriage, still birth, bleeding in pregnancy and hypertensive disorders, levels of education, marital status, and current age, as the predictor variables** | **7** |
| Data sources/ measurement | 8* | For each variable of interest, give sources of data and details of methods of assessment (measurement). Describe comparability of assessment methods if there is more than one group  Response: Information regarding cognitive impairment as the primary outcome variable was gathered using the Montreal Cognitive assessment tool which assessed the presence of cognitive impairment and dementia (MoCA) [8]. There are two versions of the MoCA tool; MoC-A and MoCA-B. In this study, we adopted MoCA-B due to its cultural sensitivity and more relevant to our sample. The MoCA-B is a rapid screening instrument which assesses cognitive domains of orientation, short-term memory, visuo-spatial abilities, attention/concentration, language, and aspects of executive functioning. This tool possesses strong psychometric properties with good test-retest reliability and internal consistency (0.83) [41, 42], and it has been validated in sub-Saharan Africa i.e., in South Africa [42] and in East Africa [41]. This version was specifically created for screening patients with low education and low literacy levels [42]. The total score for MOCA-B tool ranges from 0-30 suggesting with a cut-off point >25 suggesting normal cognitive functions, moderate CI (18-25), mild (11-18) and <10 as severe cognitive impairment. | ***8*** |
| Bias | 9 | Describe any efforts to address potential sources of bias. **Response.**  **Data was collected by trained medical doctors and one Psychologist. This team had been trained in research and data collection using standardized tools.** | **14** |
| Study size | 10 | Explain how the study size was arrived at.A sample size of 278 participants was determined following the methods used of sample selection by Hulley, Cummings, Browner, Grady, Newman [36].n = N*X / (X + N – 1), where, X =  114 Zα/22 *p*(1-p) / MOE^2^, and Zα/2 is the critical value of the normal distribution at α/2 (e.g. for a 115 confidence level of 95%, α = 0.05, and the critical value is 1.96). MOE is the margin of error, p is the sample proportion, and N is the population size. | **5** |
| Quantitative variables | 11 | Explain how quantitative variables were handled in the analyses. If applicable, describe which groupings were chosen and why  Response: **Data were secured in a cabinet under lock and the key was only accessible by the lead investigator. Data were reviewed for completeness, missing fields identified and filled at the end of each data collection day. We generated data into excel spread sheet using Kobo Toolbox (Harvard Humanitarian Initiative, Cambridge, Massachusetts, United States of America). From excel, cleaned data was entered into STATA 17.0 (Stata Corp, College Station, Texas, USA). We computed standard statistics to summarize characteristics of the sample as well as the prevalence of dementia within our sample. The association between various social-demographic factors was estimated using logistic regression** | **7** |
| Statistical methods | 12 | (*a*) Describe all statistical methods, including those used to control for confounding. **Response: Descriptive statistics and their corresponding percentages were used to describe the participants demographic characteristics.** **Logistic regression analysed the association between different variables and cognitive functions** | **8** |
|  |  | (*b*) Describe any methods used to examine subgroups and interactions. **Response:** **Confidence interval and p values were used to determine the level of significancy** |  |
|  |  | (*c*) Explain how missing data were addressed. **Response:** **During cleaning process, the missing participants with missing data were excluded.** |  |
|  |  | (*d*) If applicable, describe analytical methods taking account of sampling strategy- **NA** |  |
|  |  | (*e*) Describe any sensitivity analyses- **None** |  |
| Results | | |  |
| Participants | 13* | Report numbers of individuals at each stage of study—eg numbers potentially eligible, examined for eligibility, confirmed eligible, included in the study, completing follow-up, and analysed **Responses: Overall, 280 women aged between 40 to 90 years with a mean age of (53.5[10.6]) participated in this study.** | **8** |
|  |  | (b) Give reasons for non-participation at each stage  Response: **Our sampling procedure was non probability in nature (consecutive), we had originally hoped to assess 278 participants but got more as the number kept snowballing up to 280** |  |
|  |  | (c) Consider use of a flow diagram. Response: **Due to the nature of our sampling procedure, we could not consider the use of flow diagram.** |  |
| Descriptive data | 14* | (a) Give characteristics of study participants (eg demographic, clinical, social) and information on exposures and potential confounders: Response: **Overall, 280 women aged between 40 to 90 years with a mean age of (53.5[10.6]) participated in this study. Nearly all the participants in this study were married 83.6% (234/280). Almost a quarter of our participants 29% (80/280) had experienced more than one pregnancy complication**. | **9** |
|  |  | (b) Indicate number of participants with missing data for each variable of interest; Response. **We had 3partcipnats missing information on social demographic questions and these were removed from the analysis** |  |
| Outcome data | 15* | Report numbers of outcome events or summary measures | **10** |
| Main results | 16 | 1. Give unadjusted estimates and, if applicable, confounder-adjusted estimates and their precision (eg, 95% confidence interval). Make clear which confounders were adjusted for and why they were included;   **Response:** To estimate for the strength of different factors that were associated with cognitive functioning, multiple logistic regression between were used. At a bivariate level (Unadjusted analysis) results revealed that place of delivery, age of women and education level had a statistically significant association with cognitive functioning (p<0.05). At a multivariate level (Adjusted analysis) results revealed that participants who delivered from health facilities had less chances of developing cognitive impairment compared to those who delivered from other places other than a health facility (OR=0.31,95%CI:0.16-0.60, p=<.001). Participants who were more than 65 years had substantially high odds of developing cognitive impairment compared those who are 65 and below (OR=2.94; 95%CI: 0.96-9.04), p=0.06). | **11** |
|  |  | (*b*) Report category boundaries when continuous variables were categorized; Response: **Although some variables such as age, education in years and cognitive impairment could have been analysed as continuous variables, scientifically, they would give us meaningful results when categorized** |  |
|  |  | (*c*) If relevant, consider translating estimates of relative risk into absolute risk for a meaningful time period **Response: Not relevant and already explained in the text above** |  |
| Other analyses | 17 | Report other analyses done—eg analyses of subgroups and interactions, and sensitivity analyses: Response: **No further analysis was performed** | **n/a** |
| Discussion | | |  |
| Key results | 18 | Summarise key results with reference to study objectives: **Response: Our results indicate high prevalence of cognitive impairment among the women with history of pregnancy complications in rural southwestern Uganda. Specific forms of cognitive impairment observed in our sample included; mild (45%), moderate (31%) and severe (4%). Being above the age of 65 had more odds of developing cognitive impairment than being below the age of 65 while giving birth from a health facility and having higher education positively correlated with cognitive functioning** | **12** |
| Limitations | 19 | Discuss limitations of the study, taking into account sources of potential bias or imprecision. Discuss both direction and magnitude of any potential bias  **Response: The prevalence of cognitive impairment in our study is slightly higher than the study of Kintu and colleagues [6] in south western Uganda which focused on urban residents with high literacy levels following traumatic brain injury. Our results are also in line with a study done among elder women rural China [43]. One possible reason for this high prevalence of cognitive impairment is that, previous studies with a low prevalence used different tools other than the MOCA-B to asses cognitive decline [7, 44] while other studies were not clear on the methods for cognitive assessment [18].Another possible explanation for this high prevalence in our sample is that the prevalence of cognitive decline and dementia in southwestern Uganda where our sample was derived has also been reportedly to be high [6, 7, 12].The variation between our findings and findings from other previous studies could be due to the fact that these studies recruited participants who were older than 65 years and hence did not cater for the possibility of early onset of cognitive impairment [45]. The question that remains**  **unanswered, therefore, is whether the high prevalence of cognitive impairment is related to the history of pregnancy complications or whether there are other moderating factors that need to be considered. We therefore suggest that the specific aspects of risk and resilience of early onset of dementia in the context of African rural setting be investigated more closely in future studies. In line with previous studies, we found an association between increased age and cognitive impairment [43]. The finding that increasing age is associated with cognitive impairment is not surprising. For example, one recent study among the survivors of traumatic brain injury in south western Uganda found that cognitive impairment was associated with older age [6] while another study in the same community found that increase in age was associated with dementia [7]. One important finding in our study was that there was a significant positive association between higher educational levels and cognitive functioning. We found that the cognitive function of participants within our sample with 7and above years of education performed better on cognitive on the MOCA-B than those with lower education. Our finding about the correlation between education and cognitive performance is further confirmed by other previous studies that have showed that educational enrichment in early life acts as a defence against cognitive impairment in late life and buffers the development of cognitive impairment [46, 47].Similarly, our results confirm he cognitive reserve hypothesis which seems to propose that education has a protective effect that buffers cognitive impairment in the general population[48, 49]. For example, one study found out that cognitive reserve delayed decline in cognitive functions among the old people before the onset of Alzheimer's disease [48].**  **We propose that the area of cognitive reserves in form of enhancing education levels be emphasized by clinicians and policy maker. The finding that having delivered babies from a health facility positively correlated with good cognitive functioning is in agreement with the results of a number of studies that found out that patients with psychosis who combined both biomedical and traditional healing systems, experienced abettor outcome health [50-52]. Furthermore, our study population was largely comprised of women with low literacy levels. Low literacy levels were the biggest modifiable risk factor for development of cognitive impairment according to a systematic review in sub-Saharan Africa that looked for the risk factors of cognitive decline in the region [53]. We suggest that the likelihood of developing dementia among women with a history of pregnancy complications in low- and middle- income countries be explored further to understand the possible confounders and other predicator variables.** | **14** |
| Interpretation | 20 | Give a cautious overall interpretation of results considering objectives, limitations, multiplicity of analyses, results from similar studies, and other relevant evidence Response: In this cross-sectional study of women with a history of pregnancy complications, in rural southwestern Uganda, we found high prevalence of cognitive impairment. These findings suggest the need for further research into this phenomenon to ascertain the true picture in the population as a whole. Future studies could assess the effect of other confounders such as age at first pregnancy or first live birth, termination of pregnancy history, menopausal status and age at menopause, and iatrogenic menopause. Additionally, the comparative study between women with a history of pregnancy complications and those without is recommended to bring this phenomenon of cognitive impairment into focus. | **14** |
| Generalisability | 21 | Discuss the generalisability (external validity) of the study results **Response:** Interpretation of our findings is subject to certain limitations. First, the sample was restricted women with a history of pregnancy complications and may not be generalized to the general population samples. A second important limitation of our study is the cross-sectional design, which may not allow for causal conclusions. Thirdly, certain biases, such as recall and social desirability biases, common to retrospective designs may have affected the study findings, however, it is inevitable that such designs may be used for this kind of study. | **14** |
| Other information | | |  |
| Funding | 22 | Give the source of funding and the role of the funders for the present study and, if applicable, for the original study on which the present article is based **Response:** Research reported in this study was supported by the Fogarty International Center [U.S Department of State’s Office of the U.S. Global AIDS coordinator and Health Diplomacy (S/GAC) and the president’s Emergency plan for AIDS relief (PEPFAR)] of the National Institutes of Health Under Award Number R25TW011210 PI-Prof Obua Celestino | **15** |

*Give information separately for exposed and unexposed groups.

**Note:** An Explanation and Elaboration article discusses each checklist item and gives methodological background and published examples of transparent reporting. The STROBE checklist is best used in conjunction with this article (freely available on the Web sites of PLoS Medicine at http://www.plosmedicine.org/, Annals of Internal Medicine at http://www.annals.org/, and Epidemiology at http://www.epidem.com/). Information on the STROBE Initiative is available at www.strobe-statement.org.
